# Telomere dynamics and oxidative stress in Arabidopsis grown in lunar regolith simulant

**DOI:** 10.3389/fpls.2024.1351613

**Published:** 2024-02-16

**Authors:** Borja Barbero Barcenilla, Ishan Kundel, Emily Hall, Nicolas Hilty, Pavel Ulianich, Jillian Cook, Jake Turley, Monisha Yerram, Ji-Hee Min, Claudia Castillo-González, Dorothy E. Shippen

**Affiliations:** Department of Biochemistry and Biophysics, Texas A&M University, College Station, TX, United States

**Keywords:** telomeres, 8-oxoG, *Arabidopsis thaliana*, telomerase, lunar regolith simulant, oxidative stress

## Abstract

NASA envisions a future where humans establish a thriving colony on the Moon by 2050. Plants will be essential for this endeavor, but little is known about their adaptation to extraterrestrial bodies. The capacity to grow plants in lunar regolith would represent a major step towards this goal by minimizing the reliance on resources transported from Earth. Recent studies reveal that *Arabidopsis thaliana* can germinate and grow on genuine lunar regolith as well as on lunar regolith simulant. However, plants arrest in vegetative development and activate a variety of stress response pathways, most notably the oxidative stress response. Telomeres are hotspots for oxidative damage in the genome and a marker of fitness in many organisms. Here we examine *A. thaliana* growth on a lunar regolith simulant and the impact of this resource on plant physiology and on telomere dynamics, telomerase enzyme activity and genome oxidation. We report that plants successfully set seed and generate a viable second plant generation if the lunar regolith simulant is pre-washed with an antioxidant cocktail. However, plants sustain a higher degree of genome oxidation and decreased biomass relative to conventional Earth soil cultivation. Moreover, telomerase activity substantially declines and telomeres shorten in plants grown in lunar regolith simulant, implying that genome integrity may not be sustainable over the long-term. Overcoming these challenges will be an important goal in ensuring success on the lunar frontier.

## Introduction

In the pursuit of long-duration space travel and eventual colonization of celestial bodies, it will be critical to understand the adaptation of plants to these unique environments. Plants serve as essential life support systems, providing food, oxygen, carbon dioxide regeneration, and psychological benefits to astronauts (Meyers and Wyatt, 2022). Nevertheless, the successful cultivation of plants in extraterrestrial environments remains a significant challenge, as the resources required for sustained growth are scarce and transporting them from Earth is not a feasible long-term solution.

Utilization of lunar regolith as a growth substrate for plants holds promise for achieving self-sufficiency on the moon (Ming and Henninger, 1994; Wamelink et al., 2014; Wamelink et al., 2019; Paul et al., 2022). The regolith that covers the Moon’s solid bedrock consists of agglutinates formed from minerals, trapped gases, glass created by continuous meteoroid impacts, charged solar particles and solar wind (Carrier et al., 1973). Lunar regolith lacks organic matter, has minimal water content, and is deficient in essential nutrients like sodium, potassium and silicon, while being enriched in heavy metals that are toxic to plant growth (Liu et al., 2008; Braddock, 2021). In addition, this substrate is highly abrasive and potentially harmful to plant roots (Liu et al., 2008). Plants grown in lunar regolith, similar to those in spaceflight conditions and other extreme environments, activate a cadre of stress-responsive genes with the oxidative stress response being a primary target (Xie et al., 2019; Paul et al., 2022). Oxidative stress arises from an imbalance between the production of reactive oxygen species (ROS) and the capacity of antioxidant defense systems to mitigate their harmful effects. ROS, such as superoxide radicals and hydrogen peroxide, are natural byproducts of metabolic processes and environmental stressors yet also promote important developmental transitions. Abnormally high ROS levels can cause substantial damage to cellular components, including DNA, proteins and lipids, ultimately leading to cellular damage and death (Sharma et al., 2012). Thus, managing ROS through robust antioxidant mechanisms is crucial for plant health and vitality.

Telomeres consist of simple G-T rich repeated sequences that cap the ends of chromosomes to ensure that linear DNA molecules do not elicit an inappropriate DNA damage response (Lim and Cech, 2021). Telomeres also facilitate the faithful replication of terminal DNA sequences (Shay and Wright, 2019). Short telomere tracts are associated with cellular senescence and reduced viability (Stone et al., 2016; Protsenko et al., 2020). Studies in numerous organisms indicate that individuals with short telomeres have lower chances of prosperity (Stindl, 2004; Haussmann and Mauck, 2008; Bauch et al., 2014). On the other hand, elongated telomeres in humans are associated with cancer (Adam et al., 2017; Martínez and Blasco, 2017). Thus, establishing and maintaining telomere length homeostasis is important for long term survival.

Vertebrate telomeres are highly dynamic and their length and stability are influenced by a multitude of environmental factors and physiological conditions (Epel et al., 2004; Ludlow et al., 2013; Barnes et al., 2019). For example, studies in birds demonstrated that for species with longer telomeres such as *Tachycineta bicolor* there are higher survival rates (Haussmann et al., 2005), and in *Sterna hirundo*, longer telomere length is associated with reproductive success (Bauch et al., 2014). Moreover, in *Acrocephalus arundinaceus*, malaria infection is correlated with telomere length degradation, reduced lifespan and diminished reproductive success (Asghar et al., 2015). Consequently, telomere length can be viewed as a biosensor for ecological pressure.

Plant telomeres appear to be more stable than in other eukaryotic lineages. Arabidopsis maintains telomere length homeostasis in the face of heat shock, drought and high temperature (Lee et al., 2016; Campitelli et al., 2021). In addition, recent studies with plants grown on the International Space Station (ISS) indicate that telomere length is unchanged relative to Earth controls (Barcenilla et al., 2023). These findings stand in stark contrast to the fate of telomeres in astronauts and *Caenorhabditis elegans* which are grossly extended in space (Zhao et al., 2006; Garrett-Bakelman et al., 2019; Luxton et al., 2020a; Luxton et al., 2020b).

Telomere length is primarily maintained by telomerase, a ribonucleoprotein complex containing a reverse transcriptase TERT and an integral RNA template TR. Telomerase continually replenishes telomeric DNA tracts, but in plants as in mammals its activity is highly regulated and typically restricted to cells with long-term proliferation capacity (González-García et al., 2015). Telomeres, with their high G-T content, have long been proposed to be hotspots for oxidative damage in the form of 8-oxoGuanine and Thymine Glycol (Oikawa and Kawanishi, 1999; Zhou et al., 2013; Sarkar and Liu, 2016). Oxidative damage-induced lesions have the potential to compromise the structural integrity of telomeres, interfering with the formation of essential protective structures like T-loops (Wang et al., 2010; Ahmed and Lingner, 2018b; Coluzzi et al., 2019), and impeding the binding of protective proteins. Such outcomes would diminish the defensive functions of telomeres and render chromosomes more susceptible to recombination or end-to-end fusion, ultimately jeopardizing genome integrity (Opresko et al., 2005; Pinzaru et al., 2016). Telomere oxidation is also associated with replicative stress that can lead to chromosomal breakage (Coluzzi et al., 2019). While the link between telomere maintenance machinery and oxidative damage remains enigmatic, numerous studies in mammals establish this correlation (Barbero Barcenilla and Shippen, 2019). For example, the core telomerase subunits TERT and TR accumulate in mitochondria in response to oxidative stress, where they promote mitochondrial function (Santos et al., 2004; Santos et al., 2006; Ahmed et al., 2008; Haendeler et al., 2009; Cheng et al., 2018). In addition, a striking inverse correlation between telomerase enzyme activity levels and genome oxidation has been established in Arabidopsis (Barcenilla et al., 2023). Therefore, investigating telomere dynamics and oxidative damage in plants grown on lunar regolith or its simulants may provide insight into the effect of this environment on the long-term viability and genetic stability of plant populations in space.

Here we explore the growth of *Arabidopsis thaliana* on lunar regolith simulant and assess the impact of this substrate on various aspects of plant physiology, telomere dynamics and genome oxidation as measures of plant fitness. We report that pre-treatment of lunar regolith simulant with chemical antioxidants or growth of plants with genetically enhanced antioxidant activity substantially stimulates vegetative plant growth and facilitates flowering and reproductive propagation for at least two additional generation. Nevertheless, telomeres shortened in plants grown in such substrates, telomerase enzyme activity decreased, and genome oxidation increased. These findings highlight the potential of antioxidant supplementation to enhance plant fitness in extraterrestrial environments, but also raise concerns about diminishing genome stability that may hinder long-term viability of plant populations in lunar regolith.

## Materials and methods

### Growth plate preparation

48 well tissue culture plates (Genesee Cat #: 25-108) were used for initial growth. A 0.5-inch drill was used to bore a hole through the bottom of desired wells. A ~1cm X ~0.75cm piece of Grodan^®^ A-OK Stonewool starter cubes was cut and placed into the hole, leaving a small tuft of fibers on either side. Approximately 1gm of each soil type was placed into the wells and watered with watering solution through capillary action by placing the plate into a container with the liquid just covering the bottom of the container (Paul et al., 2022). Plates remained in the watering solution for ~60 sec or until the liquid had permeated the top of the soil.

### LMS-1 washing and LMS-1 recycling

An antioxidant solution was prepared by combining 0.75 mM glutathione, 0.75 mM ascorbic acid, and 0.75 mM proline, following the method outlined by El-Beltagi et al. (2020). For each gm of regolith simulant, 20 ml of washing fluid was added to achieve complete saturation, as per the procedure by Kim et al. (2021). A vessel equipped with a magnetic stir bar on a stir plate was used to maintain a full vortex column for 120 min at room temperature (Kim et al., 2021). The solution was transferred into a Nalgene Rapid-Flow Filter Unit and vacuum filtration was performed until the substrate was completely dry, either overnight or until no residual moisture remained. To recycle the LMS-1 regolith simulant, we took previously treated antioxidant regolith, which had already proven to support plant growth, and subjected it to another round of washing with the antioxidant cocktail, followed by vacuum filtration and regolith drying before replanting.

### Arabidopsis growth conditions

Seeds for Col-0 were obtained from The Arabidopsis Biological Resource Center (ABRC) at Ohio State University, stock CS28994. Seeds were surface sterilized in 2.7% sodium hypochlorite for 7 min, stratified in 4°C for three days, and transferred directly to assigned treatment of regolith simulant (LMS-1, Exolith Lab, Oviedo, FL, USA) or Earth soil (Sunshine, Mix 5, Sun Gro Horticulture, Agawam, MA, USA). Seeds were germinated under a 12-h photoperiod of an average illuminance of 5000 (+/− 250) lux attained with 3000:6500 Kelvin lights in a 1:1 ratio, and at a constant temperature of 22°C and allowed to grow under the same conditions for five weeks or until growth stopped, at which point whole plants were harvested for analyses.

### Generation of CAT2 overexpression lines

To generate *CAT2* (At4g35090) overexpression lines, pCB302-CAT2 fused with a C-terminal 2x HA epitope tag and constitutive 35S promoter was generously provided by Dr. Ping He (University of Michigan) and transformed into wild type Col-0 plants using *Agrobacterium tumefaciens* strain GV3101 via in planta vacuum infiltration (Tague and Mantis, 2006). T1 transformants with Basta resistance were selected and CAT2-HA expression was confirmed by western blotting. T2 transformants with Basta resistance were selected from transgenic lines that segregated as a single locus. T3 homozygous transgenic lines were used for further experiments.

### Statistical analysis

Statistical analyses were executed utilizing GraphPad Prism 9 for MacOS. Each experimental setup considered individual plants, with growth stages specified in figure legends. A minimum of three biological samples were included in each experiment. PCR and ELISA-based assays were performed with at least three technical replicates. Significance was assessed through p-values, deeming differences significant when below 0.05.

### DNA extraction

Genomic DNA was prepared following the standard cetyltrimethylammonium bromide (CTAB) method (Doyle and Doyle, 1987) with minor modification. A total of 0.5–1 gm of frozen plant DNA tissue was ground to a powder and mixed in a 1:1 ratio (w/v) with the CTAB lysis buffer (100 mM Tris-HCl pH 8.0, 20 mM EDTA pH 8.0, 1.4 M NaCl, 2% CTAB, 2% mercaptoethanol) and incubated at 65°C for 1 h. Phenol:chlorophorm:isoamyl alcohol (25:24:1, v/v/v) was added to the plant extract in a 1:1 ratio, and total DNA was extracted into the aqueous phase by careful mixing. The phases were separated by centrifugation at 20,000 rpm for 20 min. The aqueous phase was transferred to a new tube and DNA was precipitated for 2 h at −20°C, by adding 3M sodium acetate (1/10 aqueous phase volume) and 100% 2-propanol (2% aqueous phase volume). DNA was pelleted by centrifugation at 17,000 rpm for 20 min, washed with 70% ethanol and treated with 10 mg/mL RNase A for 30 min at 37°C.

### Telomeric DNA analysis

Single telomere analysis was performed using primer extension telomere repeat amplification (PETRA) as previously described (Heacock et al., 2004). Bulk telomere length was measure by TRF analysis using Southern blotting (Heacock et al., 2004). Telomere length was measured using the online tool, WALTER (Lyčka et al., 2021).

### Western blot analysis

Three leaf discs with a diameter of 2.5 mm were excised from the 4-week-old Arabidopsis plants expressing CAT2 C-terminal tagged with 2xHA. Three discs were frozen and ground, and 80 μl of 2x SDS loading buffer (125 mM Tris-HCl, pH 6.8, 2% SDS, 0.05 Bromophenol blue, 20% glycerol, 200 mM β-Mercaptoethanol) was added. The mixture was vortexed and boiled at 95°C for 10 min. After boiling, the mixture was centrifuged at maximum speed for 5 min. Then, 8 μl of supernatant was loaded on a 10% SDS-PAGE gel. Following electrophoresis, proteins were transferred onto a PVDF membrane (Millipore) using a Bio-Rad Trans-Blot apparatus at 25 volts for 30 min. To detect CAT2-2xHA fusion protein, a-HA- antibodies conjugated to the horseradish peroxidase (HRP; 1:2,000) was used.

### Plant measurements

Plants were gently removed from soil to ensure that the roots were not damaged and excess soil was removed from the base of the plant. Plants were individually placed on a scale to record their biomass and each ample was wrapped in aluminum foil, flash-frozen in liquid nitrogen, and stored at -80°C. Stalk height, rosette area and leaf size was calculated using ImageJ software by first taking photos of the plants, ensuring the relevant area of the plant was in-frame. Photos were uploaded to a computer running ImageJ. A scale was set using a representative known size in the plant pot. This allowed ImageJ to convert pixels to cm accurately for each photo. Once the scale was set, a line was traced from the area of interest to measure.

### Cytology

Chromosome spreads were obtained from pistils and stained with DAPI (4’,6-diamidino-2-phenylindole) as described in (Armstrong et al., 2009) with minor modifications. After 24 h of incubation in fixation solution (3 parts 100% ethanol and 1 part acetic acid), pistils were separated from the rest of the flower using watchmaker’s forceps and a needle. Two to three pistils were rinsed twice in Milli-Q water, then twice in 1X citrate buffer with 5 min incubations at room temperature. A solution of 660 μl 1X citrate buffer and 330 μl enzyme mix (1% cellulase and 1% pectolyase in 1X citrate buffer) was used for digestion. Samples were incubated for 60-90 min at 37°C in the enzyme solution and then quickly transferred to cold 1X citrate buffer to stop the reaction and incubated for at least 15 min. Pistils were placed on a sterile microscope slide with 70 μl of 60% acetic acid for 2-5 min until pistils lost their white color. A coverslip was carefully placed on top and gentle taps were applied to spread the cells. The slides with spread pistil cells were placed in liquid nitrogen for 30 sec. The coverslip was removed using a blade, and the slides were rinsed with fixative. The slides were dried for 20 min at room temperature. For chromosome staining, Drop-n-Stain EverBrite™ Mounting Medium without DAPI (23009, Biotium) was used. 18 μl of Drop-n-Stain EverBrite™ was placed on the sample, covered with a coverslip, and sealed with clear nail polish.

## Results

### Growth of A. thaliana on simulated lunar regolith is enhanced by pre-treatment with chemical antioxidants or by genetically enhanced antioxidant activity

Lunar regolith simulant is a commercially available substrate that mimics the soil composition of lunar regolith recovered from the lunar surface via prior space missions (genuine lunar regolith). Both lunar regolith simulant and more recently genuine lunar regolith have been characterized as media that support plant growth (Ming and Henninger, 1994; Wamelink et al., 2014; Paul et al., 2022). To further explore the growth and sustainability of *A. thaliana*, we performed experiments with the lunar regolith simulant LMS-1 (Long-Fox et al., 2023) supplemented with 0.125x MS watering using the conditions previously described by Paul et al. (2022) (**Supplementary Figures S1A-C**). Consistent with the former study, plants grown on LMS-1 germinated, but at a rate that was 10% of controls grown in standard Earth soil (**Supplementary Figures S1C, D**). In an attempt to improve germination efficiency, we employed a soil remediation technique to reduce the levels of heavy metals in the substrate by pre-washing with EDTA. The substrate was subsequently filtered through a Nalgene rapid-flow filter unit followed by vacuum filtration until dry (Xu et al., 2019; Kim et al., 2021) (**Supplementary Figure S1B**). This treatment did not support seed germination, and neither did supplementation of the LMS-1 with a human waste simulant (**Supplementary Figures S1C, D**).

**Figure 1:**
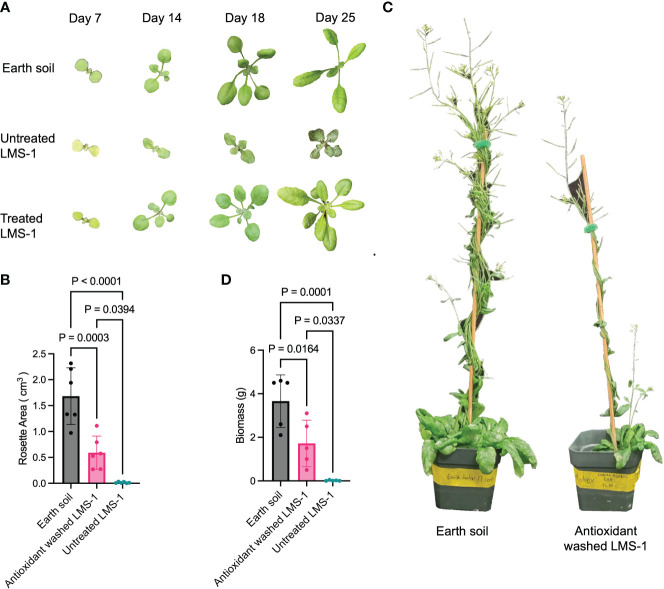
Growth of *A. thaliana* on simulated lunar regolith is increased by pre-treatment with chemical antioxidants. **(A)** Representative photos of *A. thaliana* (Col-0) grown in three conditions: Earth soil control, antioxidant washed LMS-1 and untreated LMS-1 for the times indicated. **(B)** Rosette area 25 days after germination. *P* calculated as one-way ANOVA, *n* = 6. Data displayed as mean to SD. **(C)** Photo of representative *A. thaliana* plants grown in antioxidant washed LMS-1 (right) and Earth soil (left) for 54 days. **(D)** Biomass comparison of plants grown on Earth soil, antioxidant washed LMS-1 and untreated LMS-1 after 54 days of growth. *P* calculated as one-way ANOVA, with *n* = 5 for Earth soil control and antioxidant washed LMS-1, *n* = 3 for untreated rLMS-1. Data displayed as mean to SD.

We next sought to decrease oxidative damage associated with lunar regolith by pre-washing the LMS-1 with an antioxidant cocktail consisting of 0.75 mM glutathione, 0.75 mM ascorbic acid and 0.75 mM proline (El-Beltagi et al., 2020) (**Supplementary Figures S1A-C**). Compared to untreated LMS-1, plants germinated on antioxidant-treated substrate at a rate of nearly 60% of the Earth soil control (**Supplementary Figure S1D**) and grew to yield a rosette area that was much larger than plants grown in untreated LMS-1 (0.59 cm^3^ for antioxidant LMS-1 versus 0.014 cm^3^ for untreated LMS-1) (*p =* 0.0394 calculated as one-way ANOVA, *n* = 6) (**Figures 1A, B**). Plants grown in antioxidant-treated regolith simulant for 59 days displayed an increased biomass of 1.72 gm on average compared to untreated regolith plants (ave = 0.02 gm, *p =* 0.0337 calculated as one-way ANOVA) (**Figures 1C, D**). However, relative to Earth soil controls, biomass in plants grown in antioxidant washed LMS-1 was 2-fold lower (1.72 gm vs 3.7 gm) (*p =* 0.0164 calculated as one-way ANOVA) (**Figures 1C, D**).

**Figure 2:**
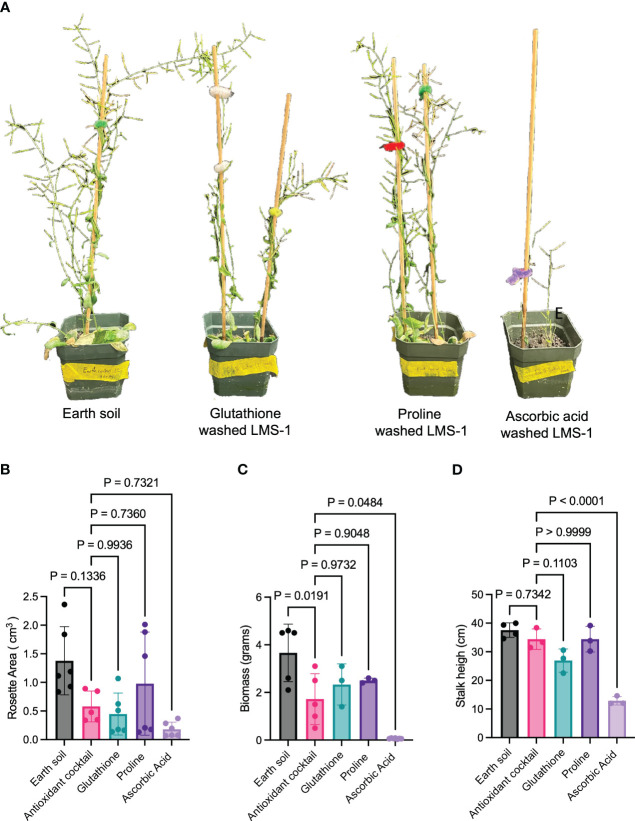
Germination of *A. thaliana* in LMS-1 requires glutathione and proline. **(A)** Representative photos of Col-0 at 59-days old after growth in Earth soil, antioxidant cocktail washed LMS-1 and plants grown in LMS-1 washed with either ascorbic acid, glutathione or proline. **(B)** Rosette area measurements 25 days after germination in the Earth soil control, antioxidant washed LMS-1 and LMS-1 washed separately in the three components. *P* calculated as one-way ANOVA. *N* = 6 for Earth soil control, glutathione, proline and ascorbic acid. *N* = 5 for complete antioxidant cocktail. **(C)** Biomass measurements for 59-day-old plants grown on Earth soil, antioxidant washed LMS-1, and LMS-1 with the three individual components of the antioxidant cocktail. *P* calculated as two-way ANOVA. *N* = 5 for Earth soil and antioxidant cocktail and *n =* 3 for glutathione, proline and ascorbic acid. **(D)** Stock height measurements for 59-day old plants grown on Earth soil, antioxidant washed LMS-1 and LMS-1 treated with individual components of the antioxidant cocktail. *P* calculated as one-way ANOVA. *N* = 4 for Earth soil and *n =* 3 for antioxidant cocktail, glutathione, proline and ascorbic acid. Data displayed as mean to SD for every graph.

Subsequent experiments indicated the individual components of the antioxidant cocktail had different effects on plant growth relative to the complete antioxidant cocktail and the untreated LMS-1 (**Figure 2**). Compared to antioxidant-treated LMS-1, proline-treated substrate yielded plants with a similar rosette area (ave_proline_ = 0.98 cm^3^, ave_antioxidant_ = 0.58 cm^3^, *p* = 0.74 calculated as one-way ANOVA) (**Figure 2B**), biomass (ave_proline_ = 2.50 gm, ave_antioxidant_ = 3.66 gm, *p* = 0.90 calculated as two-way ANOVA) (**Figure 2C**) and height (ave_proline_ = 34.41 cm, ave_antioxidant_ = 34.40 cm, *p* > 0.99 calculated as one-way ANOVA) (**Figure 2D**). Likewise, plants treated with 0.75 mM glutathione displayed a similar growth-promoting ability was those treated with the complete antioxidant cocktail in terms of biomass (ave = 2.33 gm, *p* = 0.97 calculated as two-way ANOVA) (**Figure 2C**), rosette area (ave = 0.44 cm^3^, *p* = 0.99, calculated as one-way ANOVA) (**Figure 2B**), and plant height (ave = 26.91 cm, *p* = 0.11 calculated as one-way ANOVA) (**Figure 2D**). Conversely, 0.75 mM ascorbic acid failed to stimulate the same extent of plant growth compared to full antioxidant treatment (**Figure 2A**), leading to reduced plant height (ave = 12.88 cm, *p* < 0.0001 calculated as one-way ANOVA) (**Figure 2D**) and biomass (ave = 0.07 gm, *p* = 0.048 calculated as two-way ANOVA) (**Figure 2C**), but similar rosette area (ave = 0.18 cm^3^, *p* = 0.73 calculated as one-way ANOVA) (**Figure 2B**). These data support the conclusion that exogenous application of antioxidants can enhance plant growth on lunar regolith simulant.

In parallel with the chemical antioxidant treatment, we used a genetic approach to increase the level of antioxidant activity of *A. thaliana in vivo* by ectopically expressing the antioxidant enzyme Catalase 2 driven by the powerful CaMV 35S promoter (*35S::CAT2*). Ectopic Catalase 2 expression was verified using western blotting (**Supplementary Figure S2A**). *35S::CAT2* line 11 was chosen for further analysis. This line achieved an 89% germination rate on LMS-1 supplemented with the antioxidant cocktail, similar to the wild type Col-0 on this substrate (**Supplementary Figures S2B, C**). However, *35S::CAT2* showed a remarkable 77% germination rate on untreated LMS-1, compared to only 22% for Col-0 (**Supplementary Figures S2B, C**). Similar to Col-0 grown on untreated LMS-1 (**Figure 1**), *35S::CAT2* plants that were germinated on untreated LMS-1 were not viable past 20-days of growth. We also analyzed plants completely devoid of CAT2, *cat2* null mutants. These plants had a germination rate of only 15% on antioxidant-treated LMS-1 and completely failed to germinate on the untreated LMS-1 substrate (**Supplementary Figures S2B, C**). We conclude that enhancing antioxidant mechanisms through chemical or genetic means can substantially stimulate *A. thaliana* growth on lunar regolith simulant.

### Antioxidant treatment enables plants grown in lunar regolith simulant to transition to the reproductive phase and produce viable progeny

**Figure 3:**
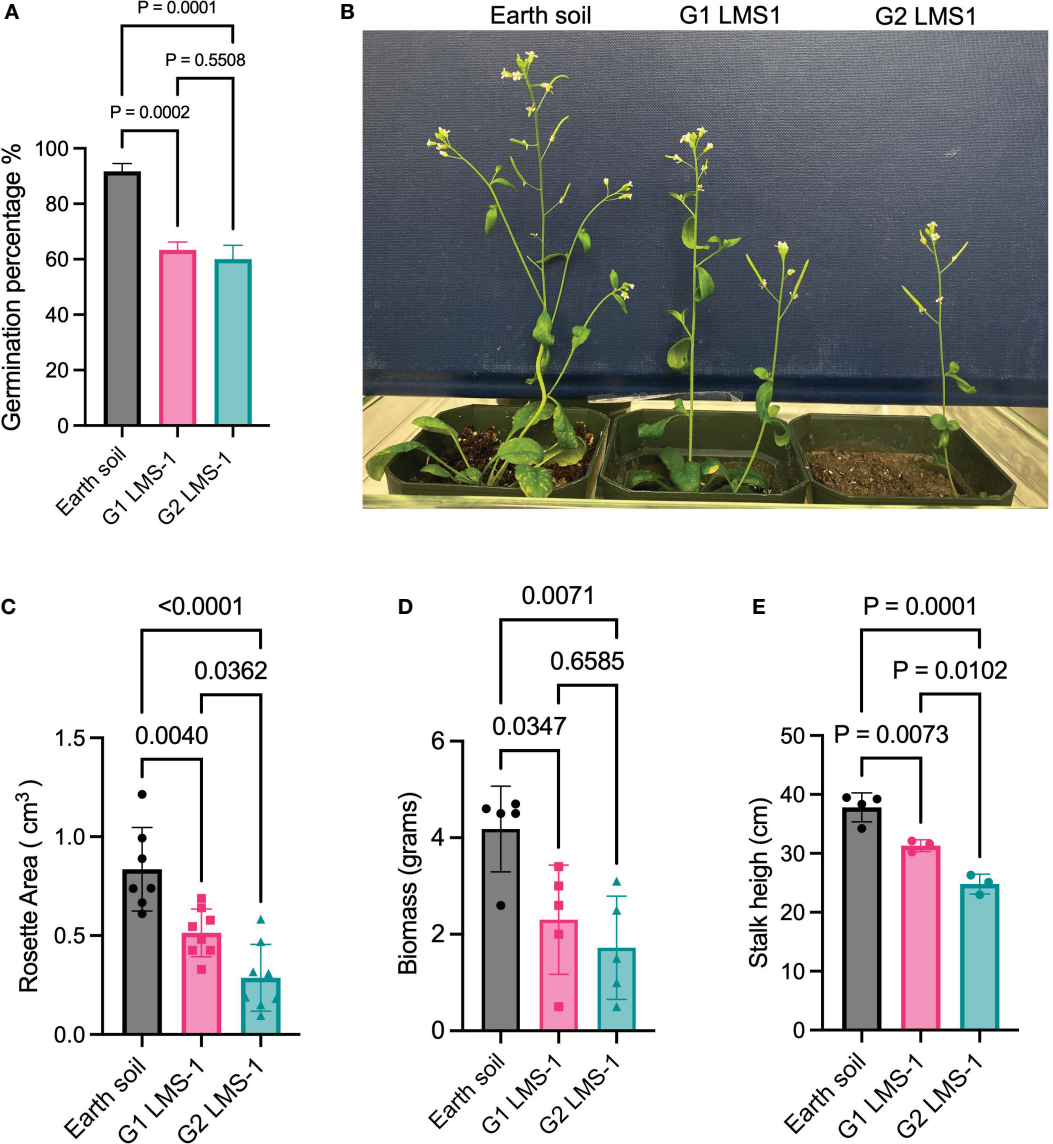
LMS-1 pre-wash with antioxidant cocktail enables multiple generations of viable, but smaller progeny. **(A)** Germination percentage of Earth soil control, first generation (G1) LMS-1 (pre-treated with complete antioxidant cocktail) progeny and second generation (G2) LMS-1 (pre-treated with complete antioxidant cocktail) three days post imbibition. *P* calculated as one-way ANOVA, with *n* = 36. **(B)** Representative photos of 34 day-old *A. thaliana* grown on Earth soil or antioxidant washed LMS-1plants and their G1 and G2 progeny **(C)** Rosette area measurements for plants grown in Earth soil, G1 Antioxidant washed LMS-1, and G2 antioxidant washed LMS-1 23 days after germination. *P* calculated as ordinary one-way ANOVA. *N =* 8 for G1 and G2 LMS-1 and *n* = 7 for Earth control. **(D)** Biomass measurements for Earth soil, G1 antioxidant washed LMS-1, G2 antioxidant washed LMS-1 54 days post-germination. *P* calculated as ordinary one-way ANOVA with *n* = 5. **(E)** Stalk height measurements for plants grown in Earth soil, G1 Antioxidant washed LMS-1 G2 antioxidant washed LMS-1 54-days after germination. *P* calculated as ordinary one-way ANOVA. *N =* 4 for Earth soil and *n* = 3 for G1 and G2 antioxidant washed LMS-1 plants. Data displayed as mean to SD for every graph.

The establishment of interplanetary colonies will necessitate continuous plant growth across multiple generations. Our experiments and previous studies (Paul et al., 2022) indicate that while plants can germinate in untreated LMS-1, they arrest in vegetative growth without producing a germline. Strikingly, plants germinated in antioxidant-treated LMS-1 (generation 1 or G1) can successfully transition to the reproductive phase and produce viable seeds for generation 2 (G2) progeny (**Figures 3A, B**). A 60% germination rate was observed in both G1 LMS-1 and G2 LMS-1 (*p* = 0.5508 calculated as one-way ANOVA), although both sets of plants displayed a lower germination rate than the Earth soil control (*p <* 0.0005 calculated as one-way ANOVA) (**Figure 3A**). We collected seeds from G2 LMS-1 plants and determined that they successfully germinate, indicating that a third generation of viable plants may be possible. Analysis of G1 LMS-1-grown plants 23 days post germination revealed a rosette area that was reduced two-fold (ave_G1 =_ 0.51 cm^3^) compared to its Earth soil grown plants (ave_EC_ = 0.83 cm^3^, *p* = 0.0040 calculated by one-way ANOVA) (**Figure 3C**). The rosette area was four-fold less than for G2 antioxidant-washed LMS-1 plants compared to earth soil (ave_G2 =_ 0.28 cm^3^, *p* < 0.0001 calculated as one-way ANOVA) (**Figure 3C**). Biomass measurements taken 54 days post-germination also revealed a reduced net weight of both G1 (ave = 2.38 gm, *p* = 0.0347 calculates as one-way ANOVA) and G2 plants (ave= 1.72 gm, *p* = 0.0071 calculated as one-way ANOVA) relative to the Earth soil control (ave = 4.05 gm) (**Figure 3D**). Plant height of G2 antioxidant-washed LMS-1 plants was less (ave = 24.807 cm) than either G1 antioxidant-washed LMS-1 plants (ave = 31.311 cm, *p* = 0.01 calculated as one way ANOVA) or the Earth soil control (ave = 37.801 cm, *p* = 0.0001 calculated as one way ANOVA) (**Figure 3E**). These findings indicate that although plants can be grown for successive generations in antioxidant-treated lunar regolith simulant, generational growth leads to progressively reduced plant size and biomass, implying that inclusion of antioxidants in the lunar regolith simulant is insufficient to promote robust growth over successive generations.

### Decreased telomere length and telomerase activity in Arabidopsis grown on lunar regolith simulant

**Figure 4:**
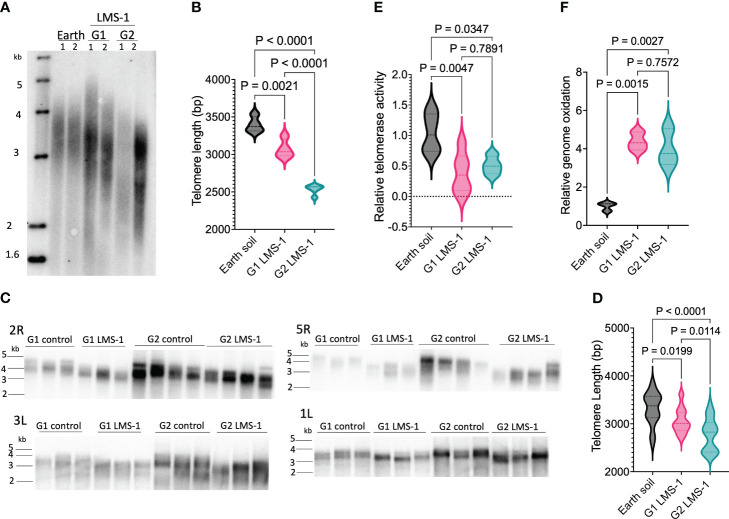
Telomere shortening in plants grown for successive generations in LMS-1. **(A)** Bulk telomere length analysis by TRF conducted on 54 day-old plants grown on Earth soil and G1 and G2 plants grown on Earth control antioxidant washed LMS-1. Each lane represents an independent biological full plant tissue replicate with *n* = 4. Data shown for 2 biological replicates. Additional results are shown in **Supplementary Figure S3**. **(B)** Mean telomere length determined from TRF data (in this figure and in **Supplementary Figure S3**) analyzed by WALTER. Data are shown as violin plots. *P* calculated as ordinary one-way ANOVA. **(C)** Individual telomere length measured by PETRA conducted on 54 day-old *A. thaliana* plants grown on earth control or antioxidant washed regolith simulant for chromosome arms 2R, 3L, 5R and 1L. Each lane represents an independent biological replicate. *N =* 12 for G1 control and G1 LMS-1, and *n =* 14 for G2 control and G2 LMS-1. **(D)** Mean telomere length determined from PETRA data analyzed by WALTER. *P* calculated as ordinary one-way ANOVA. Data displayed as violin plots. **(E)** Relative telomerase activity measured by Q-TRAP on 30 day-old plants grown on earth control or antioxidant washed regolith simulant with *n* = 6 for Earth control and G1 lunar, and *n* = 4 for G2 lunar. For each biological replicate, 3 technical replicates were performed. *P* calculated as one-way ANOVA. Data displayed as violin plots. **(F)** Relative genome oxidation measured as genomic 8-oxoG content for 54 day-old plants grown on Earth soil or antioxidant washed LMS-1. *P* calculated as one-way ANOVA. *N* = 3 and for each plant biological replicate, three technical replicated were performed. Data displayed as violin plots.

Given previous studies showing that telomere length is maintained in Arabidopsis subjected to a variety of different environmental exposures (Lee et al., 2016; Campitelli et al., 2021; Barcenilla et al., 2023), we investigated whether telomere length would be similarly preserved in plants grown in lunar regolith simulant. Plants grown in untreated LMS-1 did not produce sufficient biomass for telomere length analysis, and therefore we measured telomeres in G1 and G2 plants grown in antioxidant-treated LMS-1. We used two strategies to assess telomere length: Terminal Restriction Fragmentation (TRF) analysis which determines bulk telomere length and Primer Extension Telomere Repeat Amplification (PETRA) which assesses telomere length on individual chromosome arms (Heacock et al., 2004). Average telomere length was quantitated by WALTER software (Lyčka et al., 2021). TRF analysis revealed telomeres of plants grown in Earth soil were an average of 3396 bp in length which, as expected, falls within the wild type size range of 2-5 kb for the Col-0 accession (Shakirov and Shippen, 2004) (**Figures 4A, B**; **Supplementary Figure S3A**). Unexpectedly, however, telomeres in G1 plants grown on antioxidant-treated LMS-1 were shorter, measuring 3073 bp for a net decrease of 323 bp relative to the Earth soil control (ave = 3396 bp, *p* = 0.0021 calculated by one-way ANOVA) (**Figures 4A, B**). When the progeny of these plants were propagated to the next generation (G2), telomere length was even shorter (2560 bp) for a further loss of 513 bp relative to G1 plants (*p = <* 0.0001 calculated by one-way ANOVA) (**Figures 4A, B**, **Supplementary Figure S3A**). PETRA conducted on the telomeres from chromosome arms 5R, 3L, 2R and 1L likewise revealed telomere shortening. The average telomere length for Earth soil grown plants for these chromosome arms was 3317 bp (**Figures 4C, D**). In contrast, PETRA showed G1 antioxidant-washed LMS-1-grown plants had telomeres of 3063 bp on average (*p* = 0.02 calculated by one-way ANOVA), while for G2 antioxidant-washed LMS-1 plants the average telomere length was 2777 bp (*p* < 0.0001 calculated by one-way ANOVA) (**Figures 4C, D**). Thus, our PETRA data are consistent with the results of bulk telomere length analysis and indicate that relative to Earth soil grown plants, telomeres in plants grown in lunar regolith simulant for consecutive generations decline by approximately 300 bp in G1 and by an additional 500 bp in G2.

To confirm that telomere shortening was not an artifact of antioxidant treatment, we tested if plants grown on Earth soil that was pre-washed with the antioxidant cocktail undergo telomere shortening. Plants were grown for 10 days on this substrate and then telomere length was assessed by PETRA. We found no significant difference in telomere length between samples grown in untreated Earth soil (3449 bp) versus Earth soil treated with antioxidant cocktail (3617 bp, *p* = 0.08 calculated by unpaired *t-*test) (**Supplementary Figures S3B–D**). These data argue that some component(s) in the lunar regolith simulant are responsible for telomere shortening.

Telomere shortening can occur through a variety of mechanisms including loss of telomerase enzyme activity (Fitzgerald et al., 1999; Riha et al., 2001). To investigate whether telomerase was impaired by growth on lunar regolith simulant we measured telomerase enzyme activity using quantitative telomere repeat amplification protocol (Q-TRAP) (Kannan et al., 2008). We observed a significant reduction in relative telomerase activity, with G1 antioxidant-washed LMS1-grown plants registering only 0.39-fold the amount of telomerase activity of the Earth soil controls (*p* = 0.0047 calculated by one-way ANOVA) (**Figure 4E**). Similarly, the level of telomerase activity in G2 antioxidant-washed LMS-1 plants was half that of plants grown in Earth soil (0.51 fold, *p* = 0.035 calculated by one-way ANOVA), and not statistically different from the G1 plants (*p* = 0.79 calculated by one-way ANOVA) (**Figure 4E**).

Telomere shortening can also occur if chromosome ends become deprotected leading to nucleolytic digestion. However, we did not observe evidence of DNA degradation: PETRA products were represented by sharp bands (**Figure 4C**), indicating telomere tracts were largely intact. In addition, analysis of mitotically dividing cells in the pistils of antioxidant-washed LMS-1 grown *A. thaliana* did not reveal evidence of anaphase bridges that would be consistent with the end-to-end chromosome fusions (Heacock et al., 2004) (**Supplementary Figure S4**).

### Elevated genome oxidation in plants grown in lunar regolith simulant

Given the elevated oxidative stress response associated with plants grown in lunar regolith (Paul et al., 2022), we asked if the genome of plants grown in LMS-1 accumulates oxidative damage by assessing levels of genomic 8-oxoG. Compared to plants grown in Earth soil, *A. thaliana* grown in antioxidant washed LMS-1 displayed a 4-fold increase in genome oxidation for both G1 and G2 plants (*p_G1 lunar_* = 0.0015, *p*_G2 lunar_ = 0.0027 calculated by one-way ANOVA) (**Figure 4F**). There was no difference in genome oxidation levels between G1 and G2 antioxidant-washed LMS-1 plants (*p* = 0.76 calculated by one-way ANOVA) (**Figure 4F**).

**Figure 5:**
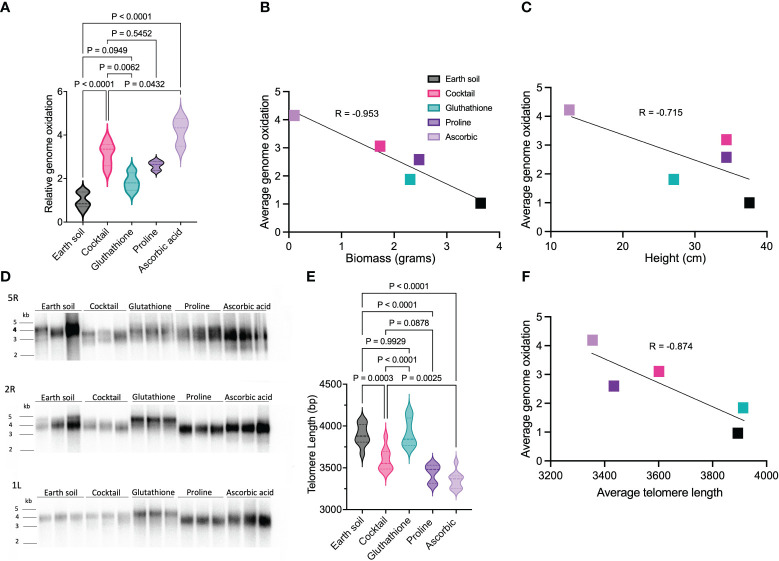
Effect of antioxidant treatment on genome oxidation and telomere length for plants grown on LMS-1. **(A)** Relative genome oxidation measured as genomic 8-oxoG content for 54 day-old plants grown on Earth soil, antioxidant cocktail washed LMS-1, and LMS-1 washed individually with glutathione, proline or ascorbic acid. *N* = 5 for Earth soil and antioxidant cocktail washed LMS-1, and *n* = 3 for LMS-1 washed with glutathione, proline or ascorbic acid. For each biological replicate (individual plant), three technical replicated were performed. *P* calculated as two-way ANOVA. Data displayed as violin plots. **(B)** Inverse relationship between average biomass and average genome oxidation for plants grown on Earth control, antioxidant washed LMS-1 and LMS-1 washed with glutathione, proline or ascorbic acid. Two-tailed Pearson’s correlation *r* = -0.953, xy pairs (*n*) = 5. **(C)** Inverse correlation between average height and average genome oxidation for plants grown on Earth soil, antioxidant washed LMS-1, or LMS-1 washed with glutathione, proline or ascorbic acid. Two-tailed Pearson’s correlation *r* = -0.715, xy pairs (*n*) = 5. **(D)** Individual telomere length analysis measured by PETRA conducted on 54 day-old plants grown on Earth soil, antioxidant cocktail washed LMS-1, and LMS-1 washed with glutathione, proline and ascorbic acid. Results are shown for chromosome arms 2R, 5R and 1L. Each lane represents an independent biological replicate (individual plant) with *n =* 9. **(E)** Mean telomere length determined from PETRA data analyzed by WALTER. *P* calculated as ordinary one-way ANOVA. Data displayed as a violin plot. **(F)** Inverse correlation between average telomere length and average genome oxidation in plants grown on Earth soil, antioxidant cocktail washed LMS-1 and LMS-1 washed with glutathione, proline or ascorbic acid. Two-tailed Pearson’s correlation *r* = -0.874, xy pairs (*n*) = 5.

We also assessed the impact of individual components within the antioxidant cocktail on genome oxidation, since each component had a distinct effect on plant growth. LMS-1-grown plants treated with ascorbic acid (which exhibited smaller plant size, **Figure 2**) had the highest 8-oxoG levels compared to the Earth soil control (ave = 4.2 fold increase, *p* < 0.0001 calculated by two-way ANOVA) and the antioxidant cocktail (*p* = 0.043 calculated by two-way ANOVA) (**Figure 5A**). Conversely, for plants treated with proline, genome oxidation was similar to that of plants treated with the complete antioxidant cocktail (*p* = 0.55 calculated by two-way ANOVA). Interestingly in the case of glutathione treatment, relative genome oxidization was lower when compared to antioxidant cocktail full treatment (*p* = 0.0062 calculated by two-way ANOVA) and similar to Earth soil (ave = 1.82 fold change, *p =* 0.095 calculated as two-way ANOVA). These observations support the notion that antioxidants can ameliorate genome oxidation in lunar regolith simulant-grown plants. Notably, the extent of oxidative damage was inversely correlated with plant biomass (*r* = -0.95 calculated as Pearson correlation between plant biomass and 8-oxoG) (**Figure 5B**) and height (*r* = -0.72 calculated as Pearson correlation between plant height and 8-oxoG) (**Figure 5C**).

The addition of glutathione to the lunar regolith simulant was remarkably effective for maintaining telomere length. Telomere length in plants grown in this substrate (3917 bp) was the same as for Earth soil controls (3890 bp, *p* = 0.99 calculated as one-way ANOVA) (**Figures 5D, E**). Conversely, ascorbic acid treatment led to the most substantial reduction in telomere length compared to Earth soil (3353 bp, *p* < 0.0001 calculated as one-way ANOVA), as well as when compared to plants subjected to full antioxidant treatment (*p* = 0.0025 calculated as one-way ANOVA). Proline treatment resulted in telomeres that were shorter than plants grown in Earth soil (3438 bp, *p* < 0.0001 calculated as one-way ANOVA), but a similar length as in plants subjected to the full antioxidant cocktail (*p* = 0.088 calculated as one-way ANOVA) (**Figures 5D, E**). We conclude that inclusion of glutathione in the regolith simulant is sufficient to effectively preserve telomeres at the same length as in plants grown in Earth soil. By contrast, proline-treated LMS-1 yielded plants with telomeres of a length similar to those grown in the full antioxidant cocktail, whereas ascorbic acid treatment failed to replicate the effects of the complete antioxidant cocktail. Further analysis revealed a strong negative correlation between telomere length and 8-oxoG measured for each of the antioxidant treatments (*r* = -0.87 calculated as Pearson correlation) (**Figure 5F**). The variations in telomere length and overall size in LMS-1-grown plants, particularly the decrease in telomere length with ascorbic acid treatment and its recovery with glutathione, correlate with their impacts on the level of oxidative DNA damage. The relatively high 8-oxoG content in ascorbic acid-treated plants is accompanied by telomere shortening and smaller plant sizes. In contrast, glutathione appears to effectively counteract genomic oxidative stress, preserving telomere length and sustaining healthier plant growth. Taken together, these data indicate that genomic DNA oxidation is substantially increased in plants grown in lunar regolith simulant, consistent with the transcriptional activation of an oxidative stress response (Paul et al., 2022). However, antioxidant pre-wash, particularly with glutathione, can mitigate this damage.

Finally, since oxidative stress levels can affect organelle function (Gonzales-Ebsen et al., 2017), we monitored organellar function by assessing the abundance of chloroplasts and mitochondria in plants cultivated on LMS-1 using PCR to amplify genes encoded by mitochondria (*cox1, rps4*, and *atp1*), chloroplasts (*psbA*, *clpP* and *ndhH*) (Weihe, 2014) or nuclei (*RpoTp* and *RpoTm*) as a control. We detected no substantial difference in the amount of mitochondrial and chloroplast DNA in G1 plants grown in antioxidant treated LMS-1 (0.77 fold-change_mito_, 0.63 fold-change_chloro_), G2 plants grown in treated LMS-1 (1.06 fold-change_mito_, 1.23 fold-change_chloro_) and the Earth soil controls (1.01 fold-change_mito_, 1.12 fold-change_chloro_) (**Supplementary Figure S5**). We conclude mitochondrial and chloroplast DNA levels are not impacted by cultivation in antioxidant-treated LMS-1 substrate.

### Reused lunar regolith simulant leads to more robust growth than fresh LMS-1

**Figure 6:**
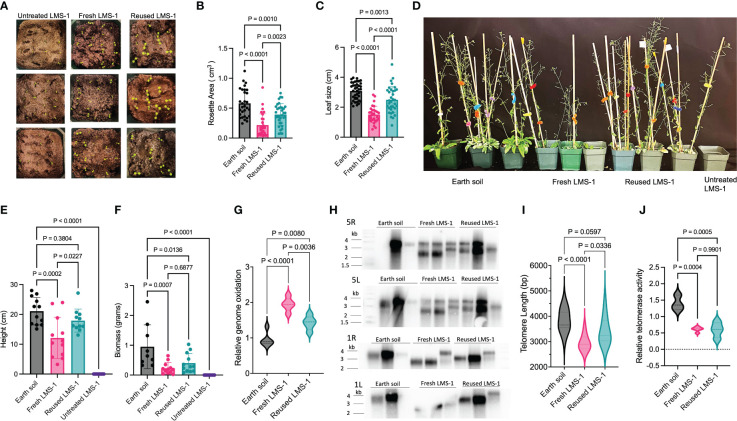
Enhanced growth on antioxidant-treated reused LMS-1 relative to fresh substrate. **(A)** Representative photos of plants germinated on untreated fresh LMS-1, antioxidant cocktail treated fresh LMS-1 and antioxidant treated reused LMS-1 at 3-day post germination. **(B)** Rosette area comparison between Earth soil, antioxidant washed LMS-1 and reused antioxidant washed LMS-1 18 days post-germination. *P* calculated as ordinary one-way ANOVA. Plot displayed as mean with SD. **(C)** Leaf size comparison between Earth soil, antioxidant washed fresh LMS-1 and reused antioxidant washed LMS-1 18 days post-germination. *P* calculated as ordinary one-way ANOVA. Plot displayed as mean with SD. **(D)** Representative photos of plants grown in Earth soil, untreated fresh LMS-1, antioxidant washed fresh LMS-1 and reused antioxidant washed LMS-1 at 34-day post germination. **(E)** Stalk height comparison between plants grown in Earth soil, untreated fresh LMS-1, antioxidant washed LMS-1 and reused antioxidant washed LMS-1 at 34-day post germination. *P* calculated as ordinary one-way ANOVA. Plot displayed as mean with SD. **(F)** Biomass comparison between plants grown in Earth soil, untreated fresh LMS-1, antioxidant washed LMS-1 and reused antioxidant washed LMS-1 at 34-day post germination. *P* calculated as ordinary one-way ANOVA. Plot displayed as mean with SD. **(G)** Relative genome oxidation measured as genomic 8-oxoG content for 34-day-old plants grown on Earth soil, antioxidant washed LMS-1 and reused antioxidant washed LMS-1. Data displayed as violin plot with *p* calculated as ordinary one-way ANOVA, *n* = 5 with three technical repeats performed per biological sample. **(H)** Individual telomere length analysis measured by PETRA conducted on 34-day-old plants grown on Earth soil, antioxidant washed fresh LMS-1 and lunar regolith simulant, and antioxidant washed reused LMS-1. Data are shown for chromosome arms 5L, 5R, 1L and 1R. Each lane represents data from an individual plant (biological replicate). **(I)** Mean telomere length determined from PETRA data analyzed by WALTER. *P* calculated as ordinary one-way ANOVA with *n* = 12. **(J)** Relative telomerase activity measured by Q-TRAP on 30-day-old plants grown on Earth soil, antioxidant washed fresh LMS-1, and antioxidant washed reused LMS-1 with *n* = 3 for Earth soil, *n* = 5 antioxidant washed fresh LMS-1 and *n* = 4 for antioxidant washed reused LMS-1. For each biological replicate, 3 technical replicates were performed. Data displayed as violin plots with *p* calculated as one-way ANOVA.

Recycling soil can significantly enhance plant growth (Liu et al., 2008; Kasiviswanathan et al., 2022; Wang et al., 2022). To test if reused lunar regolith simulant provided a more hospitable environment, we compared the growth of wild type *A. thaliana* in untreated, antioxidant washed, and reused antioxidant washed LMS-1 (**Figure 6A**). Plants grown on reused LMS-1 displayed visually enhanced growth with respect to antioxidant-treated fresh LMS-1 (**Figures 6A, D**). At 18 days, plants grown in reused LMS-1 displayed greater rosette area (0.014 cm^3^) compared to fresh regolith simulant (0.008 cm^3^, *p* < 0.0023 calculated as one-way ANOVA) (**Figure 6B**). Leaf size was also increased in reused LMS-1 (3.02 cm) compared to fresh regolith simulant (1.35 cm, *p* < 0.0001 calculated as one-way ANOVA) (**Figure 6C**). After 34 days, plants grown in reused regolith simulant reached an average height of 17.8 cm compared to only 12.1 cm in fresh LMS-1 (*p =* 0.023, calculated as one-way ANOVA) (**Figures 6D, E**). Notably, plant height was similar for plants grown in parallel in Earth soil (ave = 21.06 cm, *p* = 0.38 calculated as one-way ANOVA) (**Figures 6D, E**). Total biomass at 34 days decreased in reused LMS-1 (ave = 0.4 gm) compared to the Earth soil controls (ave = 0.95 gm, *p =* 0.014 calculated by one-way ANOVA), but was similar to fresh LMS-1 (ave = 0.22 gm, *p* = 0.69 calculated as one-way ANOVA) (**Figure 6F**).

We next asked if the improvement in plant growth on reused regolith simulant correlated with other molecular phenotypes. Compared to Earth soil, both fresh LMS-1 (ave = 1.94-fold change, *p* < 0.0001 calculated as one-way ANOVA) and reused LMS-1 (ave = 1.42 fold-change, *p* = 0.008 calculated as one-way ANOVA) displayed increased genome oxidation (**Figure 6G**). However, plants grown in reused regolith simulant displayed decreased genome oxidation compared to fresh LMS-1 (*p* = 0.0036 calculated as one-way ANOVA) (**Figure 6G**). Moreover, telomere shortening was not as pronounced when plants were grown in reused regolith simulant (ave = 3423 bp) compared to fresh LMS-1 (ave = 2986 bp, *p* = 0.034 calculated as one-way ANOVA) (**Figures 6H, I**). Although telomere tracts were shorter than for plants grown in Earth soil, the difference was 394 bp for plants grown in reused LMS-1 (*p* = 0.06 calculated as one-way ANOVA) and 831 bp for fresh regolith simulant (*p* < 0.0001 calculated as one-way ANOVA), compared to Earth soil (ave = 3817 bp) (**Figure 6H, I**). Telomerase activity levels were indistinguishable in plants grown in recycled regolith versus fresh regolith with both sets of plants showing 40% reduction relative to plants grown in Earth soil (*p* < 0.001 calculated as ordinary one-way ANOVA) (**Figure 6J**). Because telomeres are substantially shorter in plants grown in fresh regolith compared to the recycled substrate, these findings support the conclusion that factors besides telomerase contribute to telomere shortening in plants grown in lunar regolith simulant.

## Discussion

The remarkable discovery that Arabidopsis can germinate in both genuine and simulant lunar regolith (Ming and Henninger, 1994; Wamelink et al., 2014; Wamelink et al., 2019; Paul et al., 2022) ushers in new possibilities for establishing self-sustaining lunar missions (National Academies of Sciences and Medicine, 2023), and for promoting plant sustainability in our increasingly hostile Earth terrain. Despite this important breakthrough, plants grown in genuine lunar regolith arrest development in a vegetative state, activating a series of stress response pathways, most notably the oxidative stress response (Paul et al., 2022). In this study, we sought to examine sustainability of plants grown in lunar regolith simulant by studying the impact of antioxidant treatment on plant physiology and molecular markers of plant survivability.

Our first goal was to determine whether plant growth could be enhanced in lunar regolith simulant using a simple soil remediation strategy. We initially tried to improve the quality of the regolith simulant LMS-1 by chelating heaving metals with a pre-wash of EDTA (Xu et al., 2019), but plants failed to germinate under these conditions. We next examined the effect of pre-treating the LMS-1 substrate with an antioxidant cocktail consisting of glutathione, ascorbic acid and proline. This approach was previously shown to improve plant growth under drought stress by upregulating the antioxidant defense system and osmolyte synthesis (El-Beltagi et al., 2020). We found that the antioxidant pre-wash dramatically increased germination rate, rosette leaf area and overall biomass relative to plants grown on untreated LMS-1. Importantly, with antioxidant pre-treatment plants successfully transitioned to the reproductive phase, producing viable seeds that facilitated at least one additional generation of growth.

We also report that individual components within the antioxidant cocktail had differing effects on plant growth in LMS-1. For instance, pre-treatment with ascorbic acid was not as effective as the complete antioxidant cocktail in stimulating plant growth, but unlike plants grown in untreated LMS-1, addition of ascorbic acid allowed plants to flower and set seed. In contrast, proline and glutathione could individually replicate the growth-promoting effects of the complete antioxidant solution. Exogenous proline application is known to enhance photosynthetic activity, increase antioxidant enzyme activities, and reduce ROS in plants under salt stress (Guo et al., 2022), while glutathione increases the activity of antioxidant enzymes, including catalase (Gul et al., 2022).

Catalase serves as a primary enzymatic defense against the oxidative damage induced by senescence, chilling, dehydration, osmotic stress, wounding, paraquat, ozone, and heavy metals (Kibinza et al., 2011). We discovered that genetic enhancement of antioxidant activity through ectopic expression of Catalase 2 (CAT2) significantly increased germination in untreated regolith simulant. Conversely, no germination was observed in a *cat2* null mutant in untreated LMS-1. Interestingly, the 35S::CAT2 plants arrested growth after 20 days and failed to flower. Thus, application of a chemical antioxidant cocktail was more effective in promoting plant growth on LMS-1. Taken together, these results offer compelling support for the proposition that reducing oxidative stress by bolstering antioxidant defenses can substantially improve the growth of *A thaliana* in lunar regolith simulant.

One of the most surprising findings from this study was the observation that telomere length declined over two generations of plant growth in LMS-1. In marked contrast to human telomeres (Bains et al., 2019; Garrett-Bakelman et al., 2019; Luxton et al., 2020a; Luxton et al., 2021), Arabidopsis telomere length is stable in the face of a broad range of abiotic environmental stressors that include heat, drought, flooding, light stress, treatment with the radiomimetic drug zeocin and spaceflight (Lee et al., 2016; Campitelli et al., 2021; Barcenilla et al., 2023). In Arabidopsis telomere shortening is associated with genetic mutation. A prime example is mutation that inactivates a core component of telomerase (Fitzgerald et al., 1999; Riha et al., 2001; Song et al., 2019). Strikingly, telomerase activity is markedly upregulated in plants subjected to chronic light exposure, zeocin exposure and spaceflight (Barcenilla et al., 2023). Telomerase induction did not occur in plants grown in LMS-1. Rather, enzyme activity declined by 40-50% relative to plants cultivated in Earth soil.

Two lines of evidence argue that this reduction in telomerase activity does not account for telomere shortening in LMS-1 grown plants. First, telomeres shorten by 250-500 bp per generation in plants null for *TERT* (Riha et al., 2001) and thus completely devoid of telomerase activity. Although this degree of telomere shortening is approximately the same as for plants grown in LMS-1, *A. thaliana* is not haploinsufficient for telomerase and plants with only 50% of the telomerase activity of wild type do not undergo telomere shortening (Fitzgerald et al., 1999). Second, telomerase activity levels were statistically indistinguishable in plants grown in recycled antioxidant washed LMS-1 versus fresh antioxidant washed LMS-1 with both sets of plants showing 40% reduction relative to plants grown in Earth soil. Nevertheless, the degree of telomere shortening in reused LMS-1 was significantly less than for plants grown fresh LMS-1. We conclude that additional factors besides telomerase contribute to telomere shortening in plants grown in lunar regolith simulant.

Telomere shortening can occur from loss of chromosome end protection, triggering exonucleolytic degradation (Song et al., 2008; Surovtseva et al., 2009; Leehy et al., 2013; Renfrew et al., 2014; Lee et al., 2016). However, telomere tracts appeared to be intact in LMS-1 grown plants as evidenced by discrete PETRA products and the absence of evidence for telomere-to-telomere fusions in mitotic chromosome spreads of these plants. A clue to a plausible mechanism of telomere shortening comes from the observation that telomere shortening in LMS-1 is accompanied by elevated genome oxidation and decreased telomerase enzyme activity. Although the reduction in telomerase activity cannot account for telomere shortening per se, it is conceivable that a decrease in telomerase activity amplifies the damaging impact of stress-induced ROS (Barcenilla et al., 2023).

Numerous studies in mammals link telomerase to the oxidative stress response (Kurz et al., 2004; Voghel et al., 2010; Fouquerel et al., 2016; Lee et al., 2017; Ahmed and Lingner, 2018a), and recent analysis of Arabidopsis mutants bearing different levels of telomerase enzyme activity reveals a strong inverse correlation between telomerase activity and genome oxidation, suggestive of a redox protective function for telomerase (Barcenilla et al., 2023). Increased genome oxidation is correlated with telomere length alterations, most notably telomere shortening (Ahmed and Lingner, 2018b). Plants cultivated in LMS-1 pre-washed with antioxidant cocktail exhibit lower levels of genomic 8-oxoG and longer telomeres than plants grown in untreated LMS-1. Moreover, treatment with glutathione alone results in even lower levels of 8-oxoG and even longer telomeres. Recent studies demonstrate that Arabidopsis telomeres are hotspots for DNA oxidation and accumulate more 8-oxoG than chromosomal bodies (Castillo-González et al., 2022). Thus, we hypothesize that the preferential buildup of oxidized bases in telomere tracts of LMS-1 grown plants triggers replication stress ultimately leading to chromosome breakage and hence telomere truncation (Ahmed and Lingner, 2018a; Fouquerel et al., 2019).

Reused regolith may also prove to be an effective means of enhancing plant sustainability. Plants cultivated on recycled LMS-1 exhibited reduced oxidative stress and less telomere shortening than plants grown in fresh LMS-1. Soil recycling is known to improve nutrient availability, optimize pH, reduce abrasiveness, increase water retention and create a microorganism flora beneficial for plant growth (Liu et al., 2008; Kasiviswanathan et al., 2022; Wang et al., 2022). Interestingly, phytoremediation can also mitigate the negative consequences of heavy metals (Yan et al., 2020). There is extensive literature linking heavy metals to telomere length changes (Pawlas et al., 2015; Zota et al., 2015; Borghini et al., 2016; Fillman et al., 2016; Wai et al., 2020; Wai et al., 2023). Since heavy metal exposure elevates ROS (Flora, 2002; Cuypers et al., 2010; Jaishankar et al., 2014), one intriguing possibility is the heavy metal content of the lunar regolith simulant (Long-Fox et al., 2023) causes increased genome oxidation, which in turn leads to telomere shortening. Recycled regolith may have less available heavy metals and hence a reduced impact on telomeres.

For long-term space missions, plant sustainability will be imperative. With antioxidant wash, we demonstrated Arabidopsis could be propagated for at least two generations on LMS-1 substrate. However, fitness was clearly decreasing through the generations. Telomerase activity diminished and genome oxidation increased in the G1 progeny. Although these values did not change in G2, telomeres were even shorter in this generation. Persistent stress has been reported to lead to progressive telomere shortening (Kurz et al., 2004). We speculate that sustained telomere shortening in plants grown on lunar regolith simulant is the result of ongoing oxidative stress. If unabated telomere shortening will ultimately compromise genome stability and thus additional intervention will be required to sustain plant viability in this setting.

In summary, our study underscores the potential advantages of antioxidant supplementation in lunar regolith environments as a means to alleviate oxidative stress and foster plant growth. We further propose that the successful establishment of lunar farming will require a comprehensive understanding of the intricate interplay between redox homeostasis and telomere maintenance mechanisms. Such information will be important for transitioning from model organisms like Arabidopsis to the cultivation of crop plants for sustainable agricultural systems on the moon and beyond.

## Data availability statement

The raw data supporting the conclusions of this article will be made available by the authors, without undue reservation.

## Author contributions

BB: Conceptualization, Data curation, Formal analysis, Investigation, Methodology, Supervision, Writing – original draft, Writing – review & editing. IK: Data curation, Formal analysis, Investigation, Writing – review & editing. EH: Formal analysis, Investigation, Methodology, Writing – original draft. NH: Data curation, Investigation, Writing – original draft. PU: Writing – review & editing. JC: Methodology, Writing – original draft. JT: Writing – review & editing. MY: Writing – review & editing. JM: Methodology, Writing – review & editing. CC: Conceptualization, Writing – original draft, Methodology. DS: Conceptualization, Funding acquisition, Project administration, Resources, Supervision, Visualization, Writing – original draft, Writing – review & editing.

## Supplementary material

Supplementary Figure 1
*A. thaliana* germination in LMS-1 substrate**. (A)** Plate and rockwool set up for plant growth. **(B)** Schematic representation of LMS-1 washing technique. **(C)** Representative photos of plant germination in Earth soil, untreated LMS-1, LMS-1 washed with antioxidant cocktail, EDTA or human waste simulant (see text for details). **(D)** Germination rate for plants grown on different soil substrates as indicated.

Supplementary Figure 2 Ectopic expression of AtCAT2 leads to enhanced germination on untreated LMS-1. **(A)** Analysis of CAT2-2xHA protein expression in the T1 generation by western blot analysis. Eleven CAT2-2xHA lines with Basta resistance were obtained. CAT2-2xHA expression was detected with α-HA antibodies-HRP (1:2,000; top blot). Rubisco complex large subunit (RbsL) stained with Coomassie Brilliant Blue (CBB) was used as a loading control (bottom blot). **(B)** Representative 3-day post imbibition photos of *cat2 homozygous* mutants, the T2-11-35S::CAT2-2xHA/Col-0 (CAT2 OE) line (line 11) and wild type (Col-0) plants grown on untreated LMS-1 and antioxidant treated LMS-1. **(C)** Germination percentage for the 3-day post imbibition plants shown in panel B. For each genotype and substrate 12 seeds were planted in 3 different pots for a total of 36 seeds.

Supplementary Figure 3 The impact of antioxidant cocktail on telomere length. **(A)** Results of bulk telomere length analysis using TRF on 54 day-old *A. thaliana* plants grown on earth soil or antioxidant washed LMS-1. Each lane represents results from an independent biological replicate (plant) with *n* = 2. The other 2 biological samples can be found in . **(B)** Representative images of 10-day-old *A. thaliana* grown on Earth soil or Earth soil washed with antioxidant cocktail. The inset image in the bottom figure (white box) shows a mature plant transferred to antioxidant-treated Earth soil. **(C)** PETRA results for 10-day-old plants grown on Earth soil or Earth soil washed with antioxidant cocktail. Results are shown for telomere length analysis of chromosome arms 2R, 5R and 1L. Each lane represents an independent biological replicate (individual plant). **(D)** (top) Mean telomere length determined from PETRA data analyzed by WALTER from each chromosome arm. *P* calculated as 2-way ANOVA. Data displayed as violin plots. (bottom) Combined mean telomere length determined from PETRA data analyzed by WALTER. *P* calculated as unpaired *t*-test. Data displayed as violin plots.

Supplementary Figure 4 Analysis of mitotic figures from pistils of plants grown on Earth soil or antioxidant treated LMS-1. **(A)** Images of mitotically dividing cells in anaphase. DNA was visualized using DAPI (4’,6-diamidino-2-phenylindole). Earth soil served as a negative control and G3 *pot1a* mutants bearing critically shortened telomeres and anaphases bridges (Bose et al., 2020) served as a positive control. **(B)** Quantitation of the number of analyzed cells and the percentage of cells bearing anaphase bridges. For Earth soil, n=94 cells, antioxidant-washed LMS-1 n=102, and for G3 *pot1a* n=165. No anaphase bridges were detected wild type plants grown on either Earth soil or antioxidant-washed LMS-1. In contrast, 16.9% of the mitotically dividing cells exhibited anaphase bridges in G3 *pot1a* mutants.

Supplementary Figure 5 No change in organellar abundance for plants grown in LMS-1. **(A)** Average change in abundance of mtDNA and cpDNA was measured by qPCR for plants grown in Earth soil and G1 and G2 antioxidant washed LMS-1. Genes analyzed included mitochondrial genes *cox*1, *atp*1, *rps4*, chloroplast encoded genes *clp*1, *psb*A and *ndh*H and nuclear genome controls *RpoTp* and *RpoTm. P* calculated as two-way ANOVA. **(B)** Results of qPCR reactions for individual mitochondrial genes *cox*1, *atp*1, *rps4*, chloroplast encoded genes *clp*1, *psb*A and *ndh*H using *RpoTp* and nuclear gene *RpoTm*.
