# Supplementary figures and images for: Telomere dynamics and oxidative stress in Arabidopsis grown in lunar regolith simulant
Source: Front Plant Sci. 2024 Feb 16;15:1351613. doi: 10.3389/fpls.2024.1351613 (PMC10908177; doi:10.3389/fpls.2024.1351613)

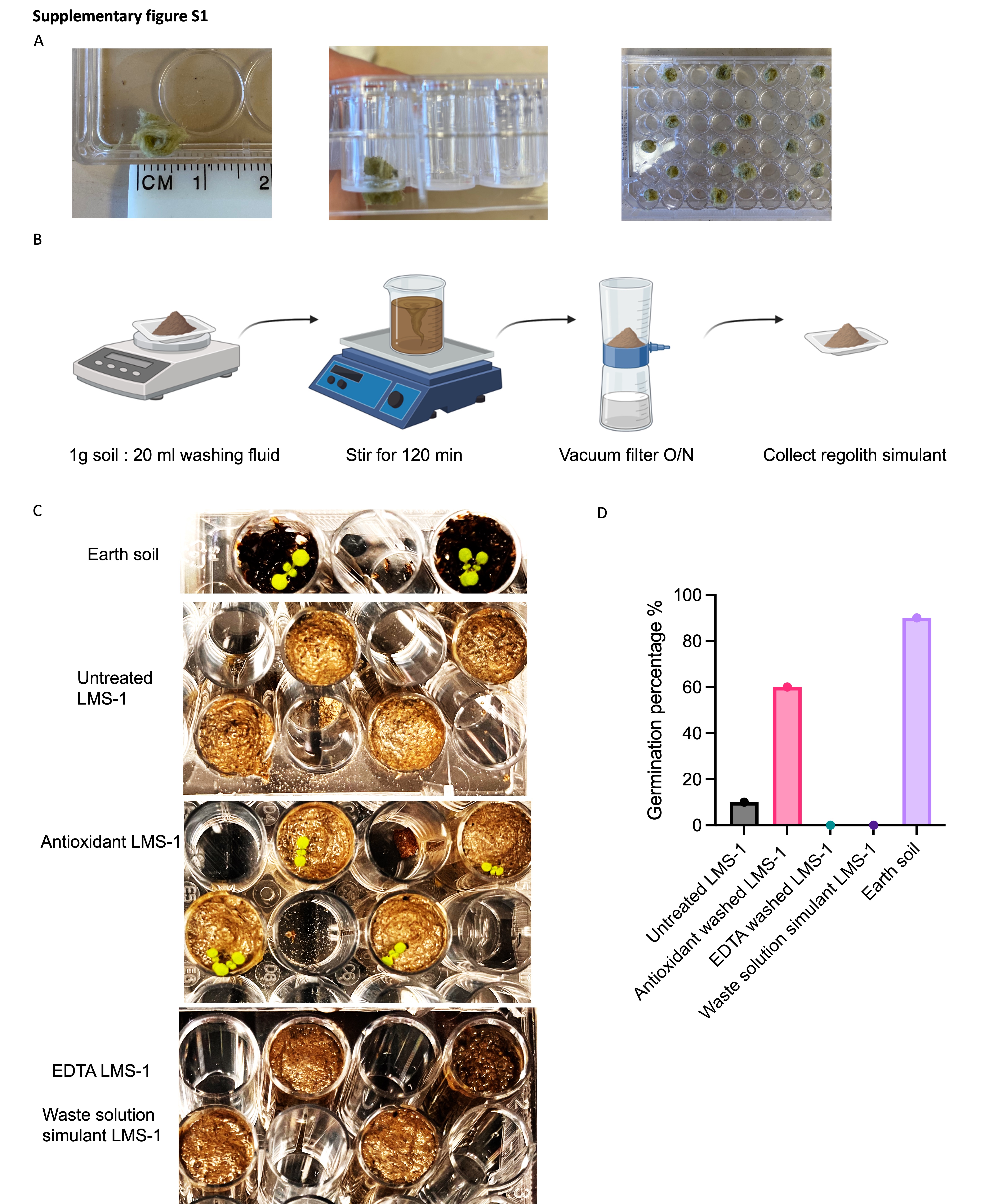

Supplement: Supplementary Figure 1 — A. thaliana germination in LMS-1 substrate. (A) Plate and rockwool set up for plant growth. (B) Schematic representation of LMS-1 washing technique. (C) Representative photos of plant germination in Earth soil, untreated LMS-1, LMS-1 washed with antioxidant cocktail, EDTA or human waste simulant (see text for details). (D) Germination rate for plants grown on different soil substrates as indicated. [file DataSheet_1.zip › Supplementary Figure 1.JPEG]

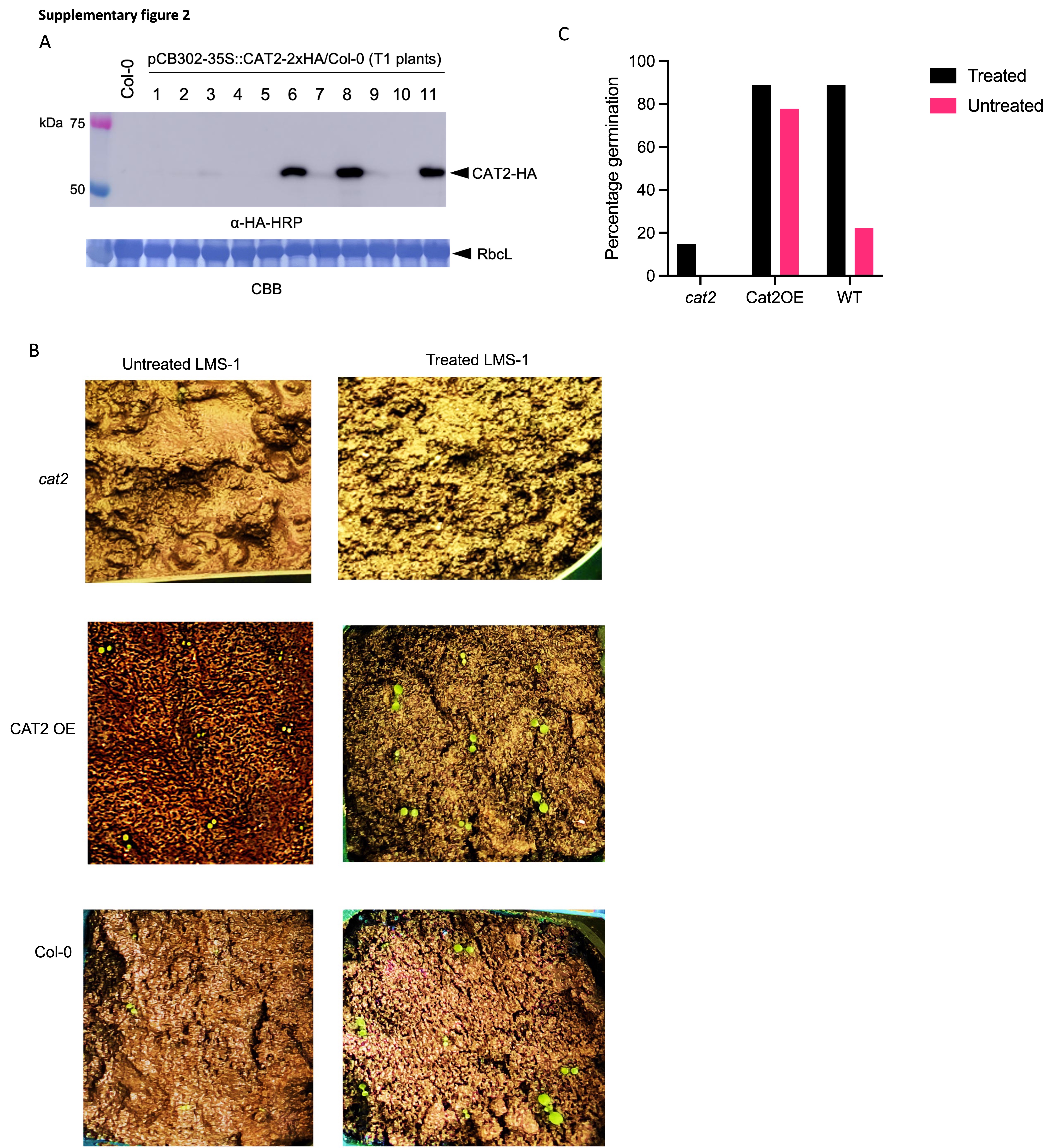

Supplement: Supplementary Figure 1 — A. thaliana germination in LMS-1 substrate. (A) Plate and rockwool set up for plant growth. (B) Schematic representation of LMS-1 washing technique. (C) Representative photos of plant germination in Earth soil, untreated LMS-1, LMS-1 washed with antioxidant cocktail, EDTA or human waste simulant (see text for details). (D) Germination rate for plants grown on different soil substrates as indicated. [file DataSheet_1.zip › Supplementary Figure 2.JPEG]

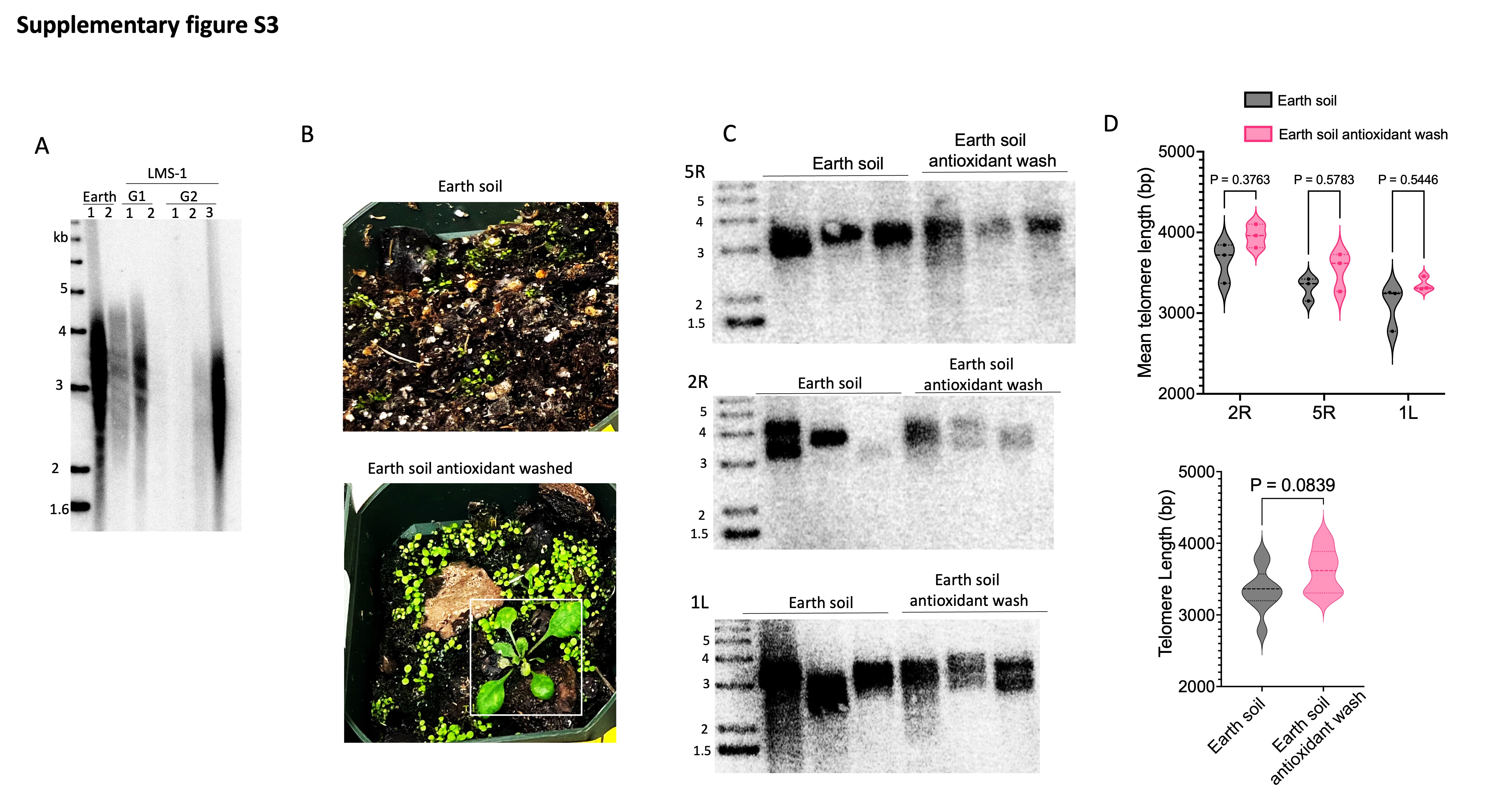

Supplement: Supplementary Figure 1 — A. thaliana germination in LMS-1 substrate. (A) Plate and rockwool set up for plant growth. (B) Schematic representation of LMS-1 washing technique. (C) Representative photos of plant germination in Earth soil, untreated LMS-1, LMS-1 washed with antioxidant cocktail, EDTA or human waste simulant (see text for details). (D) Germination rate for plants grown on different soil substrates as indicated. [file DataSheet_1.zip › Supplementary Figure 3.JPEG]

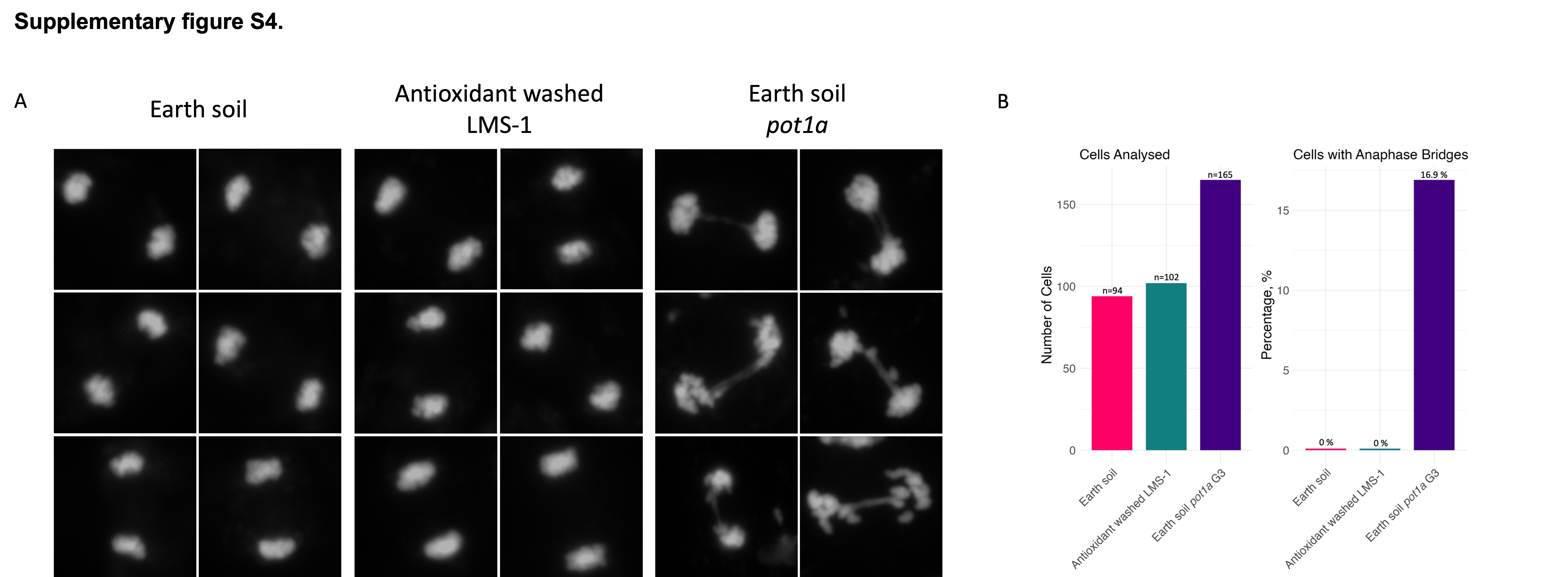

Supplement: Supplementary Figure 1 — A. thaliana germination in LMS-1 substrate. (A) Plate and rockwool set up for plant growth. (B) Schematic representation of LMS-1 washing technique. (C) Representative photos of plant germination in Earth soil, untreated LMS-1, LMS-1 washed with antioxidant cocktail, EDTA or human waste simulant (see text for details). (D) Germination rate for plants grown on different soil substrates as indicated. [file DataSheet_1.zip › Supplementary Figure 4.JPEG]

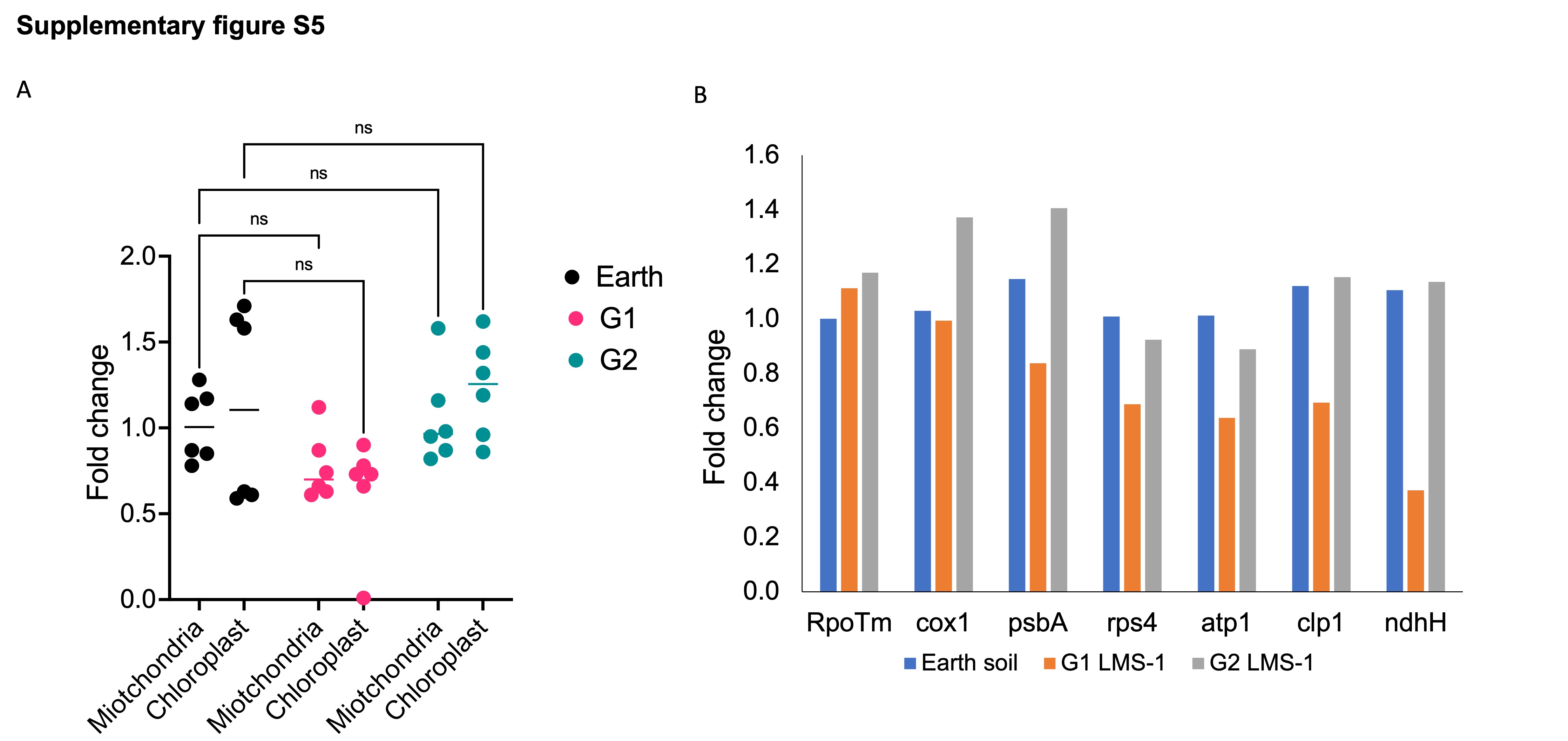

Supplement: Supplementary Figure 1 — A. thaliana germination in LMS-1 substrate. (A) Plate and rockwool set up for plant growth. (B) Schematic representation of LMS-1 washing technique. (C) Representative photos of plant germination in Earth soil, untreated LMS-1, LMS-1 washed with antioxidant cocktail, EDTA or human waste simulant (see text for details). (D) Germination rate for plants grown on different soil substrates as indicated. [file DataSheet_1.zip › Supplementary Figure 5.JPEG]
